# Antibacterial, Antioxidant Activities, GC-Mass Characterization, and Cyto/Genotoxicity Effect of Green Synthesis of Silver Nanoparticles Using Latex of *Cynanchum acutum* L

**DOI:** 10.3390/plants12010172

**Published:** 2022-12-30

**Authors:** Magda I. Soliman, Nada S. Mohammed, Ghada EL-Sherbeny, Fatmah Ahmed Safhi, Salha Mesfer ALshamrani, Amal A. Alyamani, Badr Alharthi, Safa H. Qahl, Najla Amin T. Al Kashgry, Sawsan Abd-Ellatif, Amira A. Ibrahim

**Affiliations:** 1 Botany Department, Faculty of Science, Mansoura University, Mansoura 35516, Egypt; 2 Department of Biology, College of Science, Princess Nourah bint Abdulrahman University, Riyadh 11671, Saudi Arabia; 3 Department of Biology, College of Science, University of Jeddah, Jeddah 21959, Saudi Arabia; 4 Department of Biotechnology, Faculty of Sciences, Taif University, P.O. Box 11099, Taif 21944, Saudi Arabia; 5 Department of Biology, College of Al Khurmah, Taif University, P.O. Box 11099, Taif 21974, Saudi Arabia; 6 Department of Biology, College of Science, Taif University, P.O. Box 11099, Taif 21944, Saudi Arabia; 7 Bioprocess Development Department, Genetic Engineering and Biotechnology Research Institute, City of Scientific Research and Technology Applications, Alexandria 21934, Egypt; 8 Botany and Microbiology Department, Faculty of Science, Arish University, Al-Arish 45511, Egypt

**Keywords:** *Cynanchum acutum*, cytotoxicity, genotoxicity, genomic template stability, ISSR, latex, silver nanoparticles

## Abstract

Green synthesis of nanoparticles is receiving more attention these days since it is simple to use and prepare, uses fewer harsh chemicals and chemical reactions, and is environmentally benign. A novel strategy aims to recycle poisonous plant chemicals and use them as natural stabilizing capping agents for nanoparticles. In this investigation, silver nanoparticles loaded with latex from *Cynanchum acutum* L. (Cy-AgNPs) were examined using a transmission electron microscope, FT-IR spectroscopy, and UV-visible spectroscopy. Additionally, using *Vicia faba* as a model test plant, the genotoxicity and cytotoxicity effects of crude latex and various concentrations of Cy-AgNPs were studied. The majority of the particles were spherical in shape. The highest antioxidant activity using DPPH was illustrated for CAgNPs (25 mg/L) (70.26 ± 1.32%) and decreased with increased concentrations of Cy-AGNPs. Antibacterial activity for all treatments was determined showing that the highest antibacterial activity was for Cy-AgNPs (50 mg/L) with inhibition zone 24 ± 0.014 mm against *Bacillus subtilis*, 19 ± 0.12 mm against *Escherichia coli*, and 23 ± 0.015 against *Staphylococcus aureus*. For phytochemical analysis, the highest levels of secondary metabolites from phenolic content, flavonoids, tannins, and alkaloids, were found in Cy-AgNPs (25 mg/L). *Vicia faba* treated with Cy-AgNPs- (25 mg/L) displayed the highest mitotic index (MI%) value of 9.08% compared to other Cy-AgNP concentrations (50–100 mg/L) and *C. acutum* crude latex concentrations (3%). To detect cytotoxicity, a variety of chromosomal abnormalities were used, including micronuclei at interphase, disturbed at metaphase and anaphase, chromosomal stickiness, bridges, and laggards. The concentration of Cy-AgNPs (25 mg/L) had the lowest level of chromosomal aberrations, with a value of 23.41% versus 20.81% for the control. Proteins from seeds treated with *V. faba* produced sixteen bands on SDS-PAGE, comprising ten monomorphic bands and six polymorphic bands, for a total percentage of polymorphism of 37.5%. Eight ISSR primers were employed to generate a total of 79 bands, 56 of which were polymorphic and 23 of which were common. Primer ISSR 14 has the highest level of polymorphism (92.86%), according to the data. Using biochemical SDS-PAGE and ISSR molecular markers, Cy-AgNPs (25 mg/L) showed the highest percentage of genomic template stability (GTS%), with values of 80% and 51.28%, respectively. The findings of this work suggest employing CyAgNPs (25 mg/L) in pharmaceutical purposes due to its highest content of bioactive compounds and lowest concentration of chromosomal abnormalities.

## 1. Introduction

The origin of the word nano is the Greek noun “nano”, meaning “dwarf”. Thus, nanoparticles are considered to be the primitive form of structures with sizes in the nm range. Any collection of atoms bonded together with a structural radius of 1–100 nm can be considered as a nanoparticle [1,2]. Nanoparticles display completely new or enhanced properties related to particular characteristics of size, distribution, and morphology [3,4].

There are several methods for nanoparticles to be synthesized: physical, chemical, and biological methods [5]. The weakness of using physical and chemical approaches for nanoparticles production is related to the high costs and also requiring of hazardous chemicals, so a risk of toxicity to the environment will increase and the synthesized nanoparticles are thought to be harmful [6]. To avoid utilization of harmful chemicals and eradicate the production of undesirable or baneful products, attention was turned to improve a clean, stable, benign and environment-friendly green strategy to synthesize nanoparticles [7,8]. Synthesizing of nanoparticles through plants is a relatively valuable and more profitable manner competing with using of other biological identities [9,10], Phenols, alkaloids, tannins, flavonoids, and saponins, among others, are examples of reagents that act as reductants and stabilizers during the synthesis of nanoparticles that are obtained from plant extracts [11,12]. In addition to other plants, the *Cynanchum* genus’ latex and leaf extract were employed to create silver nanoparticles (AgNPs) with antioxidant, cytotoxic, and anti-Gram-positive and anti-Gram-negative bacterial activity [13,14].

On the authority of the World Health organization (WHO), as many as 80% of the world’s people trust in plant traditional medicine for their essential healthcare requirements [15], mainly based on healing with medicinal plants [16]. Medicinal plants can be defined as any plant comprising special compounds that can be used for therapeutic aspirations and drugs production in one or more of its organs [17]. 

Latex-producing plants have been reported as a valuable medical supply in several countries due to their representative latex constituents [18]. Although herbal medicines have great benefits, there are many complications such as possibility of reducing bioavailability and little oral immersion. Nanotechnology is the promising way to overcome these shortages as nanoparticles may enhance transferring of herbal drugs for better treatment [19]. The development of nanoparticles from medicinal plants gives great chances for the enhancement of therapeutic treatments [20].

Latex is a liquid with a milky feature involving very small droplets of organic matter scattered in an aqueous medium, and so it is considered as a natural colloidal suspension [18]. Laticifers are the reservoirs of plant latex [21,22]; as latexes are established to have a defensive purpose in plants, they may have strong antimicrobial activity and thus plants can provide a good source of antimicrobial compounds [23]. The bioactive chemicals in latex showed different biological activities such as antiproliferative, vasodilatory, antimicrobial, antiparasitic [24], proteolytic [25], insecticidal [26], anti-inflammatory [27], antioxidant [28], and anticancer activities [29].

*Cynanchum acutum* L. is a latex-producing plant with high medical importance belonging to the family Asclepiadaceae. The medical importance behind several other application prospects of *Cynanchum* species allows it to serve as an important taxonomic group in the Asclepiadaceae family [30]. Crude extracts of several parts from *C. acutum* are supposed to be useful for the treatment of ulcers, representing a functional anti-ulcer agent [31]. In addition, Estakhr et al., [32] confirmed that *C. acutum* ethanol extract (200 mg/kg) exhibits anti-inflammatory actions which are related to the dose. The antimicrobial and anti-inflammatory effects of *C. acutum* were also indicated [33]. In addition, several pharmaceutically essential compounds have been identified from *C. acutum* seeds and they were supposed to have been used as sources of new and useful anticancer chemical entities [34].

Several metals have been used to synthesize nanomaterials, such as copper, zinc, titanium [35], magnesium, gold [36], and silver, for biological activities; in addition, alginate is a polysaccharide used as catalyst support [37]. Specifically, silver nanoparticles have proved to be most effective due to their specific characteristics of chemical stability, excellent conductivity, and most importantly, antibacterial, antiviral, antifungal, and anti-inflammatory features [38].

It was demonstrated that the great promise of silver nanoparticles does not prevent the occurrence of unknown risks which have not been properly estimated prior to their huge industrialized employment [39]. Proposed toxicological effects of silver nanoparticles result from the numerous ways of exposure such as domestic wastewater and chemical manufacturing, or during environment remediation efforts and crop improvement [40], in addition to the ability of AgNPs to penetrate the systemic circulation and reach several organs [41]. The negative effect of silver nanoparticles has been confirmed in several research studies such as their pathological effect in the liver by altering of liver morphology [42,43] and inflammatory effect [44]. Further, it was demonstrated that AgNPs can disturb kidney function and increase creatinine levels [45,46].

The climbing vine *C. acutum* is indigenous to Asia, Africa, and Europe. It is a plant that frequently grows in Egypt and is referred to by locals as olliq, modeid, or libbein. Insecticidal, anti-diabetic, antioxidant, antibacterial, anti-cancer, anti-inflammatory, analgesic, and antipyretic properties have been attributed to the alcoholic extract of *C. acutum* leaves [47,48]. On the other hand, the extensive exposure to nanoparticles which proceeds by the way of water, nutrition, cosmetics, medications, and drug delivery devices can lead to a broad variety of toxicological results [49]. 

In several investigations, the importance of plant latex extract in stabilizing the biogenic particles and reducing metal ions to nanoelements was underlined. Using latex extract, nitrate (AgNO3) was reduced to AgNPs having antibacterial, antioxidant, and anticancer properties [50,51]. The main objective of the current research was to determine antioxidant activity, phytochemical analysis, and antibacterial activity of different concentrations of Cy-AgNPs compared to crude latex. In addition, the lowest cytotoxic and genotoxic concentration of Cy-AgNPs able to inhibit bacterial growth was estimated. Furthermore, this work aimed to study genomic template stability using SDS-PAGE and ISSR molecular markers for different concentrations of Cy-AgNPs compared to crude latex.

## 2. Results

### 2.1. Characterization of the Synthesized Silver Nanoparticles

#### 2.1.1. UV/Visible Spectroscopy

**Figure 1:**
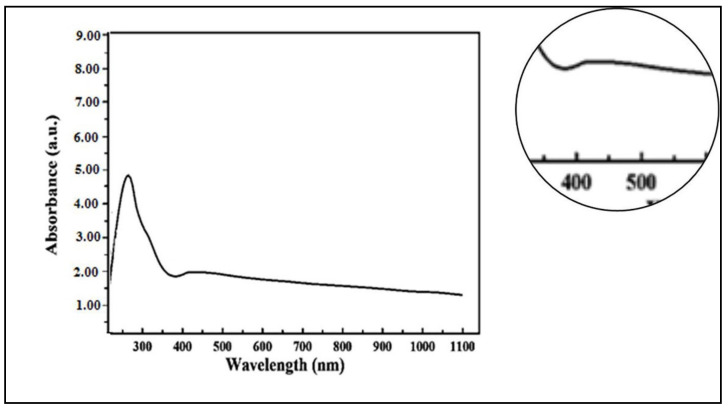
UV–vis spectra of silver nanoparticles synthesized using *C. acutum* latex extract (Cy-AgNPs); initial AgNO_3_ concentration, 1 mM.

UV–Visible spectroscopy was used to approve the reduction process of aqueous extracts by silver ions and the formation of silver nanoparticles. Silver nanoparticles started to be synthesized from *C. acutum* latex extract rapidly after 35 min of incubation. Under UV–vis spectrocopy, silver nanoparticles expressed the absorption band at λ = 432 nm, which indicates that the particle size of Cy-AgNPs was less than 100 nm [52] (Figure 1).

#### 2.1.2. Transmission Electron Microscope Analysis (TEM)

**Figure 2:**
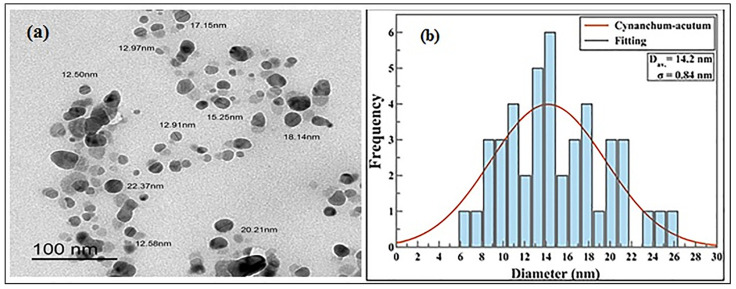
(**a**,**b**). Transmission electron micrograph of AgNPs synthesized loaded with *C. acutum* latex (Cy-AgNPs) and histogram of their size distribution.

Transmission Electron Microscopy (TEM) is a critical characterization technique for imaging nanomaterials to obtain quantitative estimation of particle size, size distribution, and morphology. Figure 2 showed TEM measurements of the synthesized nanoparticles from *C. acutum* latex and show the shape and size of the AgNPs. The greater number of the particles was spherical in shape; rare were irregular silver nanoparticles and their average size was 14.2 ± 0.84 nm.

#### 2.1.3. Fourier Transform Infrared Spectroscopy (FT-IR)

**Figure 3:**
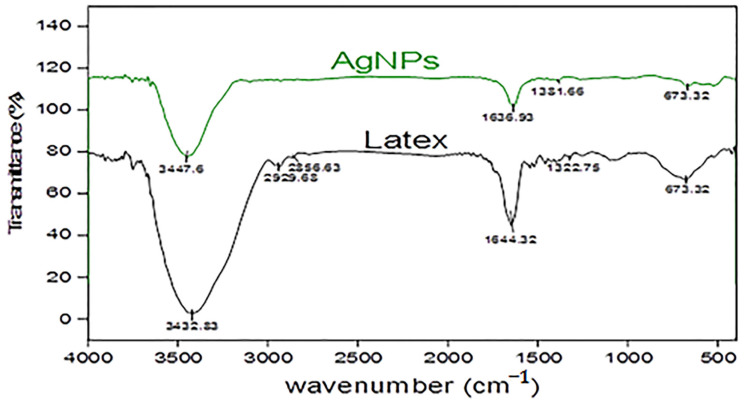
FTIR spectrum of *C. acutum* crude latex compared to that of synthesized AgNPs.

Figure 3 illustrate the FT-IR spectra of *C. acutum* latex before and after formation of silver nanoparticles; absorption bands were 3432.83 cm^−1^ and 3447.6 cm^−1^. AgNPs corresponding to O-H stretching vibration appear in the presence of alcohol and phenol. Bands at 2929.68 and 2856.63 cm^−1^ for latex arise from C–H stretching [53]. Sharp peaks located at 1644.32 and 1636.93 cm^−1^ (Cy-AgNPs) could be related to C-O or N–H bands of primary amines. Bands at 1322.75 and 1381.66 cm_−1_ (Cy-AgNPs) correspond to C–N stretching vibration of aliphatic amines [54]. The observed bands at 673.32 cm^−1^ for *C. acutum* latex and AgNPs are due to C–H bands (aromatic) [54].

### 2.2. Phytochemical Analysis

**Figure 4:**
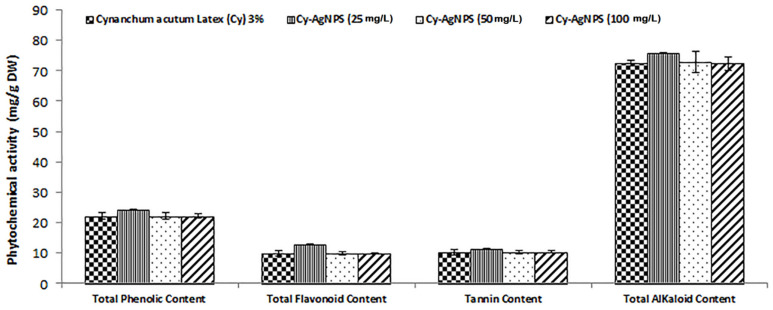
Different bioactive constituents in *C. acutum* Latex and its different nanoparticle concentrations.

Bioactive components of *C. acutum* Latex and its different nanoparticle concentrations, such as total phenolic content, total flavonoid content, tannin content, and total alkaloid content, were estimated in Figure 4. The Cy-AgNPs (25 mg/L) had the highest content of bioactive compounds with values of 24.24 mg/g DW for phenolic content, 12.86 mg/g DW for flavonoid content, 11.53 mg/g DW for tannin content, and 75.77 mg/g DW for alkaloid content. With increasing the concentration of Cy-AgNPs, bioactive compounds decreased.

**Figure 5:**
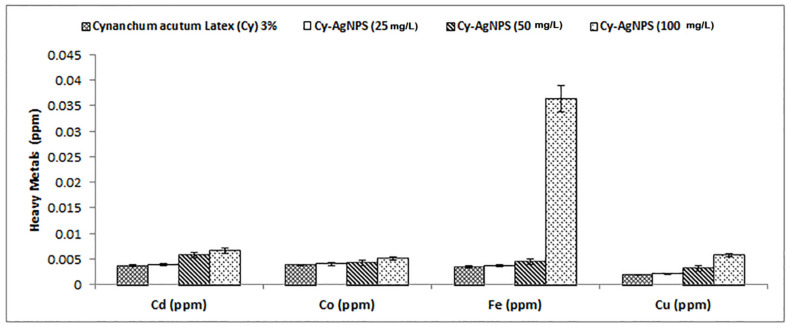
Different heavy metals in *C. acutum* Latex and its different nanoparticle concentrations.

Different heavy metals of *C. acutum* latex and different concentrations of its nanoparticles (25, 50 and 100 mg/L) were measured and illustrated in Figure 5. The highest concentrations of heavy metals were recorded in Cy-AgNPS (100 mg/L) with values of 0.0066, 0.0051, 0.0064, and 0.0057 ppm for Cd, Co, Fe, and Cu, respectively.

### 2.3. GC-MS Composition of C. acutum Latex

**Figure 6:**
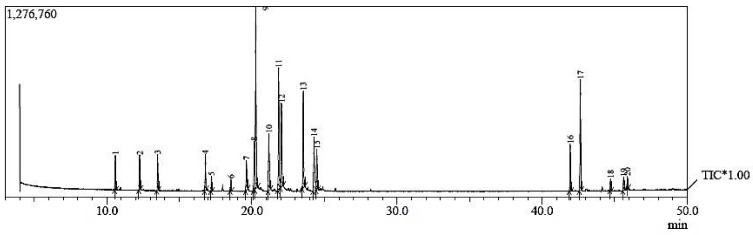
Chart of GC Mass for *C. acutum* latex.

The GC-MS analysis results of *C. acutum* latex showed bioactive compounds as listed below in Table 1. In particular, lupeol, hexadecanoic acid, neophytadiene, octadecanoic acid, and phytol showed the highest percentages of constituents present in *C. acutum* latex, which were 15.36%, 10.72%, 9.15%, 8.78%, and 6.51% respectively. The chart of GC Mass for latex of *C. acutum* is presented in Figure 6.

### 2.4. Antioxidant Activity (DPPH Scavenging Capacity (%))

**Figure 7:**
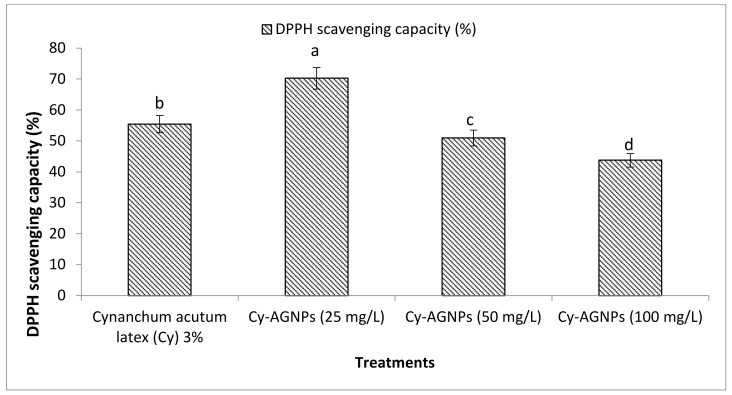
Antioxidant activity of *C. acutum* crude latex (3%) and its different AgNP concentrations using DPPH. Bars with different letters indicate significant differences between treatments at *p* ≤ 0.05. Data are expressed as the mean of three replicates ± SDs.

The antioxidant activity for *C. acutum* crude latex and different concentrations from Cy-AgNPs (25, 50 and 100 mg/L) were estimated and illustrated in Figure 7. The highest activity of DPPH was presented in Cy-AgNPS (25 mg/L) with value 70.26 ± 1.32% followed by *C. acutum* crude latex (Cy 3%) with value 55.43 ± 1.76%. Activity of DPPH decreased with increasing the concentration of Cy-AgNPs, where the lowest activity was obtained for Cy-AgNPs (100 mg/L) with value 43.76 ± 1.02%.

### 2.5. Antibacterial Activity

**Figure 8:**
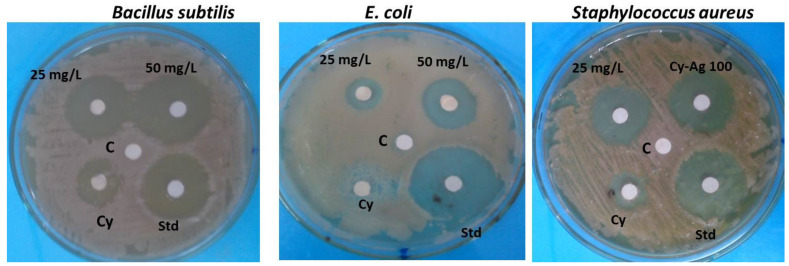
Antibacterial activity of *C. acutum* latex and different Cy-AgNP concentrations (25 and 50 mg/L) against different bacterial strains; Cy: 3% *C. acutum* latex; Std: gentamicin (10 μg) and C: negative control.

Different Cy-AgNP concentrations (25, 50, and 100 mg/L) were tested for their in vitro antibacterial effects against bacterial strains (*Bacillus subtilis*, *Escherichia coli*, and *Staphylococcus aureus*). Figure 8 shows the inhibition zones of different Cy-AgNP concentrations compared to 10 μg gentamicin standard antibiotics as the positive control and the untreated experimental control. After 24 days of incubation at 28 ± 2 °C, the highest inhibition zone was 24 ± 0.014 mm for 50 mg/L Cy-AgNPs against *B. subtilis* compared to gentamicin (22 ± 0.15). The highest inhibition zones against *E. coli* and *S. aureus* were presented for 50 mg/L Cy-AgNPs with values 19 ± 0.12 mm and 23 ± 0.015 mm, respectively.

### 2.6. Cytotoxic Effect

Cytological effects of 3% *C. acutum* latex and its different concentrations of silver nanoparticles on the mitotic cell division of *Vicia faba* root tips. The cytotoxic effect was detected at mitotic indices (MI %), phases indices (PI %), and total abnormalities (Tab %) levels and illustrated in Table 2. The highest value of (MI %) was 9.08% at 25 ppm which, was the nearest value to MI% of control (10.70%). Compared to 3% crude latex, the MI% value was 7.98%, lower than 25mg/L and higher than 50 mg/L of Cy-AgNPs. Generally, Cy-AgNPs (100 mg/L) showed the lowest mitotic index value (4.04%).

Crude latex treatment showed the value of chromosomal aberrations (25.96%). On the opposite side, 25 mg/L of Cy-AgNPs showed a significant decrease in chromosomal aberration concentrations (23.41%) compared to control and related directly with concentration, where with increasing nanoparticle concentration, the chromosomal aberrations percentage increased. The highest percentages of abnormalities were recorded at 100 mg/L (68.14%) and 50 mg/L (66.73%), compared to the control.

**Figure 9:**
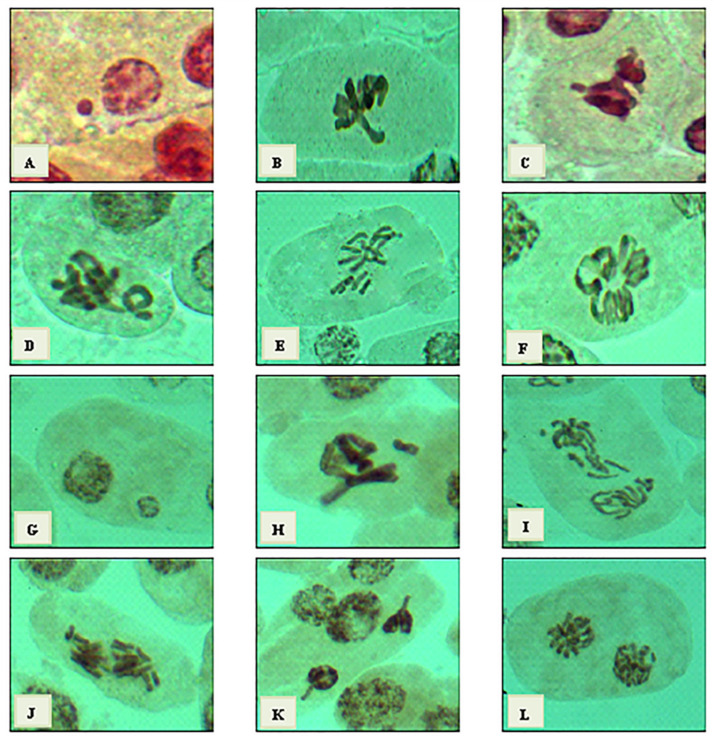
Types of mitotic abnormalities resulting from treatments of *V. faba* root tips with crude latex and Cy-AgNPS concentrations. From (**A**–**F**) crude latex, (**A**) micronucleus at interphase stage, (**B**) disturbed at metaphase, (**C**) stickiness at metaphase, (**D**) ring at metaphase, (**E**) oblique at metaphase, and (**F**) star at metaphase. From (**G**–**L**) Cy-AgNPs (25 mg/L), (**G**) micronucleus at interphase, (**H**) non-congression at metaphase, (**I**) laggard at anaphase, (**J**) disturbed at anaphase, (**K**) disturbed at telophase, and (**L**) diagonal at telophase. (X = 1000.)

**Figure 10:**
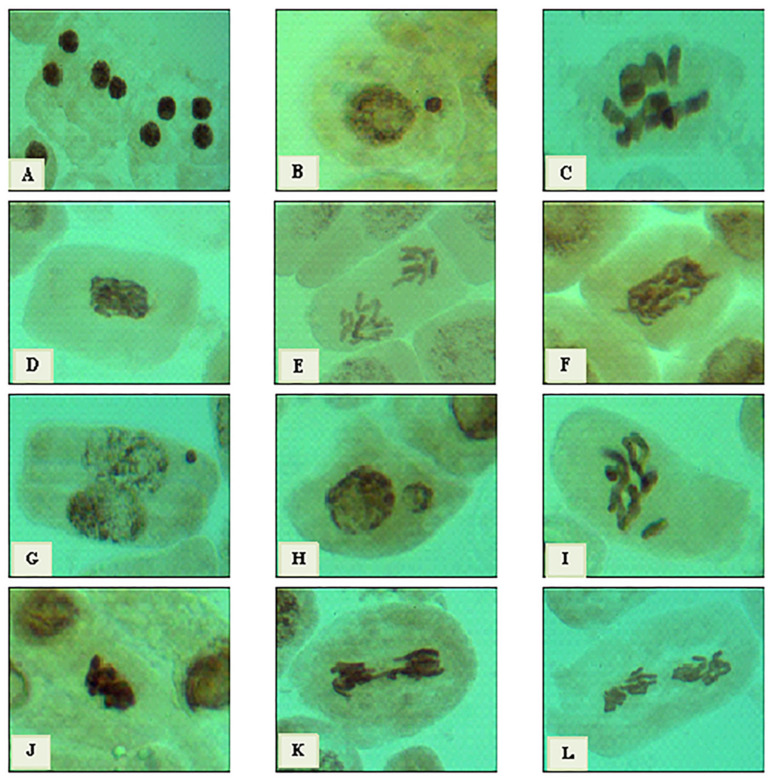
Types of mitotic abnormalities resulting from treatments of *V. faba* root tips with Cy-AgNPs. From (**A**–**F**) silver nanoparticles (50 mg/L), (**A**) binucleated cells at interphase stage, (**B**) micronucleus at interphase, (**C**) two groups at metaphase, (**D**) stickiness at metaphase, € late separation at anaphase, and (**F**) disturbed at anaphase. From (**G**–**L**) Cy-AgNPs (100 mg/L), (**G**) micronucleus at interphase, (**H**) macronucleus at interphase, (**I**) non-congression at metaphase, (**J**) stickiness at metaphase, (**K**) bridge at anaphase, and (**L**) disturbed at anaphase. (X = 1000.)

Types of chromosomal aberration appeared in treated *Vicia faba* seeds are given in Figure 9 and Figure 10. The micronucleus was recorded at interphase for all treatments and binucleated cells were observed only for the 50 mg/L Cy-AgNPs treatment. At metaphase the most common abnormalities were expressed as stickiness, non-congression, two groups, oblique, chromosome ring, fragmentation, star-metaphase, and disturbed metaphase. Late separation, bridge, and disturbed were recorded at anaphase stage. At telophase stage, bridge, diagonal, late separation, and disturbed were reported.

### 2.7. Biochemical Study Using Seed Protein Profile Electrophoresis of the Treated Vicia Faba Seeds

**Figure 11:**
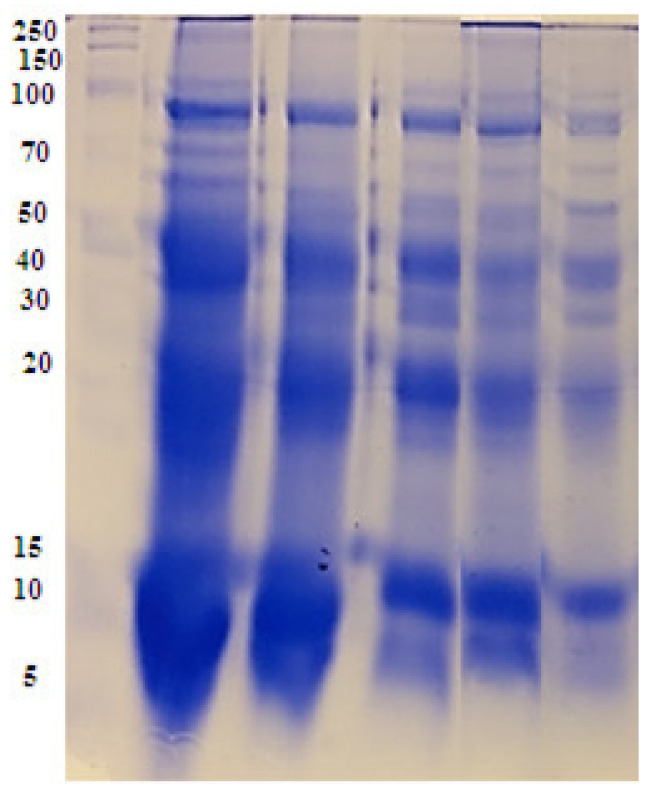
SDS-PAGE banding pattern protein in treated *Vicia faba* seeds. M: Marker, 1: control, 2: 3% latex extract, 3: Cy-AgNPs (25 mg/L), 4: Cy-AgNPs (50 mg/L), and 5: Cy-AgNPs (100 mg/L).

The protein profile in Figure 11 represents the banding patterns of the SDS-PAGE gel of treated *V. faba* seeds with 3% *C. acutum* latex and different concentrations of Cy-AgNPs. In total, sixteen bands are distinguished from the scanning of the seed protein gel of the treated *V. faba* seeds ranging between 5 and 100 KDa. All treatments caused disappearance of a band with molecular weight 17 KDa compared with the control. A band of molecular weight 19 KDa disappeared only from treated *V. faba* with Cy-AgNPs (100 mg/L); it can be used as a negative marker for this treatment. From the total thirteen bands there are ten monomorphic bands and six polymorphic bands, resulting in 37.5% polymorphism percentage among control and different treatments. The polymorphic bands divided into one unique and five non-unique bands as shown in Table 3.

### 2.8. Molecular Analysis Using ISSR Marker

**Figure 12:**
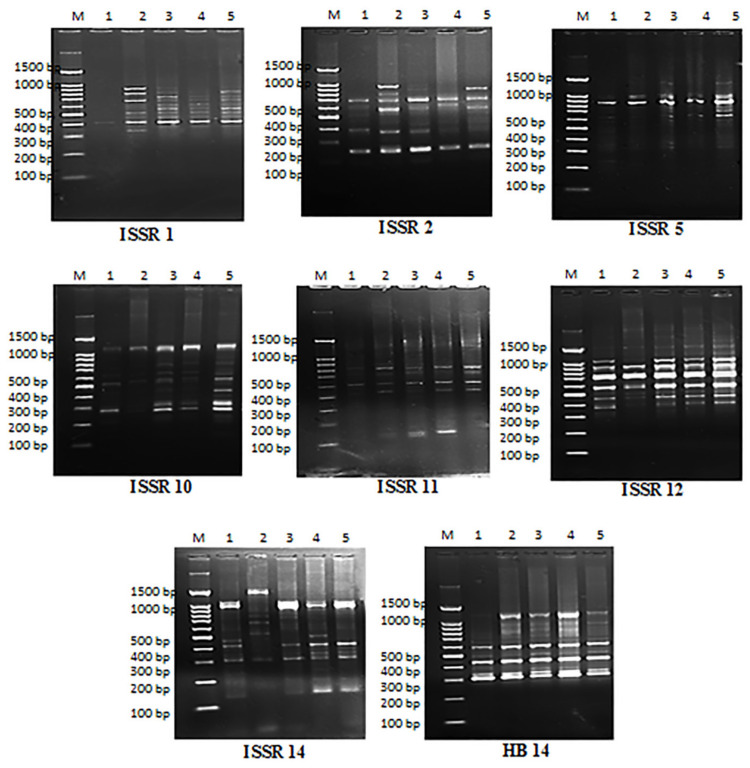
Banding profiles of ISSR for treated *Vicia faba* seeds with *C. acutum* latex and its different concentrations from Cy-AgNPs. M: Marker, 1: control, 2: Crude latex extract (3%), 3: Cy-AgNPs (25 mg/L), 4: Cy-AgNPs (50 mg/L), 5: Cy-AgNPs (100 mg/L).

ISSR analysis was operated to identify DNA alterations produced in *V. faba* cells treated with varied concentrations of Cy-AgNPs (25, 50 and 100 mg/L) and 3% latex relating to untreated sample (control). Eight ISSR primers were used and yielded banding profiles are illustrated in Figure 12. The highest percentage of polymorphism was recorded for ISSR 14 primer (92.86%) with thirteen polymorphic bands and the lowest was for ISSR 12 (30%) with seven monomorphic bands and three polymorphic bands (Table 4). 

### 2.9. Genomic Template Stability

Percentage of genomic template stability as an indicator for the changes in SDS-PAGE and ISSR was calculated and presented in Table 3 and Table 5. For SDS-PAGE, the highest percentage of genomic stability was recorded for *V. faba* treated with 25 mg/L Cy-AgNPs (80%) compared to control (100%). The percentage of genetic template stability was decreased by increasing the concentration of Cy-AgNPs; the percentage of GTS of treated *V. faba* with Cy-AgNPs (50 mg/L) showed genetic template stability percentage (73.33%) similar to GTS% for 3% *C. acutum* crude latex, where the lowest percentage of GTS recorded in Cy-AgNPs (100 mg/L) was 60% compared to control (100%).

According to the molecular results in Table 7, the maximum percentage of polymorphic bands was 35 bands for the treatment with 100 mg/L Cy-AgNPs and the minimum 29 bands for crude latex 3% treatment. Percentage of genetic template stability (GTS) showed highest value in Cy-AgNPs (25 mg/L) was 51.28% compared to control (100%); with increasing concentration of Cy-AgNPs, the percentage of GTS decreased compared to the control to 15.38% and 10.26% for 50 mg/L Cy-AgNPs and 100 mg/L Cy-AgNPs, respectively. The treatment with 3% latex showed genomic stability was 25.64% compared to control.

## 3. Discussion

The green synthesis of silver nanoparticles depends on the reduction of silver ions by phytochemicals as the primary step in the generation of nanoparticles, and these phytochemicals also play a vital role in stabilizing and fixing the shape and size of the synthesized nanoparticles [55,56]. Silver nanoparticles started to be synthesized from *C. acutum* latex extract rapidly after 35 min of incubation, similar to the results of synthesized nanoparticles from addition of *Ficus sycomorous* latex to AgNo_3_ [57]. 

UV–visible spectrophotometry is a useful technique that allows direct recognition and characterization of silver nanoparticles. Strong detected absorbance in the range 400–500 nm band known as surface plasmon resonance (SPR) resulted from the interaction between light and mobile surface electrons of silver nanoparticles [58,59]. Especially, it was supposed that recording of an absorbance band at the range of 400 nm to 450 nm represented an indicator to prove the reduction process of Ag^+^ to metallic Ag^0^ [60,61,62]. Prepared silver nanoparticles using *C. acutum* latex showed a plasmon resonance band at 432 nm similar to the green synthesized AgNPs using blackberry fruit extract which showed a broad absorption peak at λ = 435 nm [63].

Through Transmission Electron Microscopy (TEM), spherical silver nanoparticles with few irregular shapes were noticed and it was demonstrated that variability of the shape and size of nanoparticles synthesized through green approaches is very accepted [64,65]. The nanoparticles average size was 14.2 ± 0.84 nm. Thus, the *C. acutum* latex extract as a reductant yielded small AgNPs. This result is in parallel with green synthesized silver nanoparticles from *Coriandrum sativum* seed extract, which were in an average range of 13.09 nm [66]. The same outcome has been reported when *Citrullus lanatus* fruit extract was used to synthesize AgNPs with an average diameter of 17.96 nm [67]. The size and shape of nanoparticles play a critical role to be used in biotechnological and biomedical applications, and it was supposed that a smaller size is more preferred than a bigger size [68]. Dakal et al. [69] demonstrated that silver nanoparticles of spherical shape are characterized by better antimicrobial effect as it has higher surface to volume ratio to interfere with the cell walls of pathogens. The main feature of FTIR is to give an overview about the biochemical components without any disturbance in the biological sample [70].

The great matching between each latex spectrum and the AgNP spectrum of the same plant with a decrease in intensity and a slight shift in the position of peaks indicates the role of biomolecules in the formation and stabilization of silver nanoparticles. Biomolecules such as flavonoids, ketones, aldehydes, tannins, carboxylic acids, phenols, and proteins of the plant extracts are responsible for the production of AgNPs. The detected functional groups such as O-H and = C-H play a critical role in the reduction of silver ions [71]. It was reported that biological components interact with metal salts and mediate reduction processes of these functional groups [72]. The proteins could most possibly form a coat covering metal NPs (i.e., capping of AgNPs) for the prevention of agglomeration of the particles and stabilizing in the medium [73]. The detection of plant latex biomolecules in the biosynthesis of silver nanoparticles including OH and CO groups confirms their vital role in reduction and stabilization of NPs [74].

The antibacterial activity of Cy-AgNPs concentrations was determined against *Bacillus subtilis*, *Escherichia coli*, and *Staphylococcus aureus*. The highest activity with highest inhibition zone was recorded for Cy-AgNPs (50%). This effect against bacterial strains may be due to the antibacterial compounds binding to bacterial DNA after entering the inner cells through the membrane, according to a described mechanism of how the antibacterial peptides inhibit or destroy bacteria. This result is similar to research revealing that *Canarium* species’ latex was capable of acting as an antioxidant, an antibiotic, an anti-inflammatory, and a blood sugar regulator in addition to these other functions [75]. Results revealed from GC-analysis showed that *C. acutum* latex had highest content of lupeol, hexadecanoic acid, neophytadiene, octadecanoic acid, and phytol. Lupeol in many papers showed the highest antioxidant, antimicrobial, antihypoglycemic, and anti-tumor activity [76]. Hexadecanoic acid was present in *C. acutum* latex and it was also found in many plants such as *Scutellaria diffusa* aerial portion (30%), *Lycium chinense* fruits (62.89%), and *Prunella vulgaris* L. flowers (70.0%) [77]. The presence of n-alkanes such as n-tetradecane (tetradecanoic acid), n-hexadecane (hexadecanoic acid), n-nonadecane, neicosane, and n-octadecane was found in the studied latex. He [78] claimed that some alkanes have effective antibacterial properties, particularly against *Escherichia coli* and *Staphylococcus aureus*.

Plants are known to be rich in a large number of phytochemicals, which could be purified and used to cure some types of health-related diseases in addition to their nutritional value for producing dietary supplements and nutrients [79]. Evaluation of the phytochemical constituents of a medicinal plant could be considered as the most significant first step in the studies of medicinal plants [80] as it will allow great knowledge about the functional groups which enhance their medicinal properties [81]. All bioactive compounds from total phenolic, alkaloids, flavonoids and tannin content were found in Cy-AgNPs (25 mg/L) compared to *C. acutum* crude latex because NPs may differ from the bulk material and they can have improved bioactive features based on their sizes, shape, and structure [82]. In addition, NPs can induce reactive oxygen species (ROS) and secondary signaling messengers that lead to transcription regulation in plant secondary metabolism [83]. ROS and calcium ions (Ca2+) are important second messengers leading to the up-regulation of transcriptional regulators of secondary metabolites [84].

Estimation of cytotoxicity and genotoxicity has been recommended from ISO standards 10993-3 [85] in addition to 10993-5 [86] as an essential part of the evaluation process. Plants have been used to indicate environmental mutations and also demonstrate genotoxic agents [87]. Plant bioassays are preferred for being more simple, quick, efficient, and inexpensive. In addition, mutation behavior of plant cells is correlative to human and animal cells [88]. Plant models are approved to be perfect bioassays to estimate the probable genotoxicity of nanomaterials, being highly susceptible to nanotoxicity and possibly exposed to NPs by several ways such as soil, water, and air [89].

Cytotoxicity of silver nanoparticles synthesized using *C. acutum* latex was expressed through mitotic index value. The mitotic index was used as an indicator to estimate cell division frequency and has been a guideline to detect the cytotoxic effect of different agents [90,91]. By increasing silver nanoparticles’ concentration, the mitotic index was decreased. The present conclusion was in agreement with Kumari et al. [92] and Patlolla et al. [39]. It was suggested that the interference effect of highest concentration of AgNPs on the mitotic activity resulted from a delaying of cells to enter S phase (DNA synthesis) and stoppage of G2 phase; by increasing toxicant treatment, it causes cell death [93,94], which results from high concentration of Cy-AgNPs (100 mg/L). Sobieh et al. [95] suggested that the decreasing of MI was the impact of nano silver, resulting from the effect of the test agent on the growth frequency by the ability to reduce or close off the construction of metabolites required for a normal sequence of mitosis. It was clearly observed that 3% crude latex treatment leads to an arrest of metaphase stage and abortion of anaphase and telophase stages.

Metaphase arrest results from the incorrect attachment of the chromosomes to the spindle, causing inactivation of the anaphase promoting complex (APC), thus delaying the separation of sister chromatids and arresting the cell at metaphase stage and allowing anaphase to be omitted [96]. When comparing different concentrations of silver nanoparticles, anaphase and telophase stage percentages decrease with increasing concentration. The increase in metaphase index coupled with decrease in anaphase and telophase indices was related to spindle disturbance and it was supposed that the highest concentration of AgNPs had a considerable impact on spindle and therefore metaphase/anaphase transition [97]. Chromosome aberration percentage was found in the lowest concentration of Cy-AgNPS (25 mg/L) compared to *Cynanchum* crude latex; with increasing the concentration of Cy-AgNPs, the chromosome aberrations increased. This result showed the lowest concentration of Cy-AgNPs increased bioactive components to a limit, and with increasing the concentrations of nanoparticles, bioactive components decreased. So, chromosome aberrations may be decreased for the lowest concentrations of Cy-AgNPs.

Any change in the structure of chromosome is expressed as a chromosomal aberration. There are several ways to breed changes in chromosome structure such as DNA cracking, blockage of synthesis and replication of DNA [98]. Results of this study showed that the chromosome abnormalities were indicated in all treatments. Latex treatment showed the lowest value of CA. On the other hand, it showed a significant increase with increasing AgNP concentrations. Higher concentrations of AgNPs and AlO_2_NPs increased the percentage of chromosomal aberrations in *Allium sativum* root tips compared to the control [99]. The highly frequency of mitotic abnormalities may be associated with the effects of nano-silver on mitotic spindles which change the position and coordination of chromosomes at several phases of the cell cycle; silver nanoparticles also fuse the chromatin fibers, which may be related to the chromosomes’ forming stickiness and performing breaks that cause the loss of some chromosomal fragments [100]. The cytotoxic behavior of AgNPs and their ability to increase damages at chromosomes was previously shown in experiments [101].

Micronuclei were observed at interphase stage, which may be referring to spindle fibers’ malformation [102] and it was observed previously as a genotoxic effect of nano-silver [103,104]. Microscopic examinations revealed that stickiness was a major abnormality observed at metaphase stage. Sticky chromosomes were highly recorded as a genotoxic effect of AgNPs in green pea root tips [105]. The ability of silver nanoparticles to enter through the plant structure and interrupt the chemical composition of the internal components affects cell division and damages it. The toxic action of nanoparticles can be described in two different ways. The first is chemical toxicity depending on the chemical composition and ability to excrete toxic ions; secondly, tension may result from the surface, size, and/or shape of the particles [106].

SDS-PAGE was previously used in several studies to estimate the reflection of environmental stress on protein profiles [107,108]. Vannini et al., [109] indicated that some proteins affected by AgNP exposure which make protein profiles a good choice for comprehensive studies aiming to explain the molecular mechanisms highlighting the effect of AgNPs on plants. Protein profiles of treated seeds of *Vicia faba* plants showed a great variation regarding the untreated control. Generally, any alteration in protein bands between treated sample and control including disappearance of some bands may be due to the mutational potential within the regulative genes that interrupt or delay transcription [110].

DNA fingerprinting provides several effective biomarker assays in the evaluation of genotoxicity [98,99]. The inter-simple sequence repeats (ISSRs) technique is considered to be the simplest and widely used marker among the polymerase chain reaction (PCR)-based molecular techniques [111]. ISSRs are DNA-based markers based on detection of polymorphisms in inter-microsatellite loci [112]. ISSRs were successfully used to estimate the effect of heavy metals in *Hordeum vulgare* and *Pistia stratiotes* in terms of DNA [113,114].

Assessment of genomic template stability (GTS) has been used to investigate many several types of DNA destruction and mutations in animals, plants and bacterial cells [115]. GTS has been calculated as a qualitative measurement to explore genotoxicity of silver nanoparticles [116,117] and zinc nanoparticles [118]. The obtained data showed changes in band pattern by variation in the number of newly appeared bands or loss of normal bands and band intensity (increase or decrease in the intensity of amplified bands). Genomic template stability using SDS-PAGE recorded the highest for the treatment with 3% latex and the lowest for 100 mg/L silver nanoparticles; a similar observation was detected using ISSRs. Genomic template stability is connected to the frequency of DNA destruction and also the capacity of DNA to recover [119]. GTS percentage was indicated to be correlated with variations in other criteria [120]. The present results revealed that GTS% values are compatible with cytological results. All studied parameters indicate that cytotoxic and genotoxic effects of silver nanoparticles are higher than those of crude latex and that Cy-AgNPs are suitable for use at their lowest concentration.

## 4. Materials and Methods

### 4.1. Plant Material

**Figure 13:**
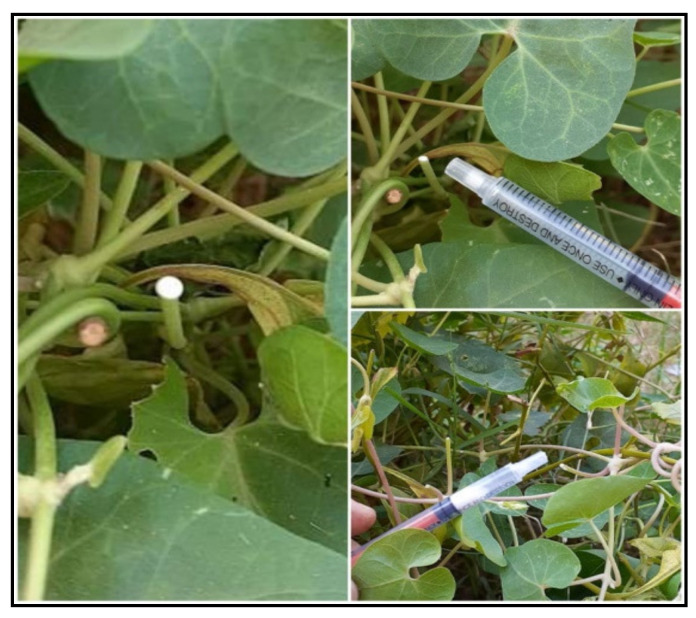
Latex collection from *C. acutum*.

Milky latex of *C. acutum* L. was collected from Mansoura University campus, Dakahlia Governorate early in the morning. The green stems were split and the white milky latex was collected in sterile bottles (Figure 13). The 3% aqueous solution of latex was prepared using distilled deionized water and stored in a freezer maintained at −4 °C until use.

### 4.2. Green Synthesis of Silver-Latex Nanoparticles

In a conical flask, 10 mL of latex (3%) was added and heated at 60 °C with continuous stirring for about 15 min using a water bath. Separately, 50 mL of AgNO_3_ solution (1 mM) was heated at 60 °C also with continuous stirring for 15 min in a water bath. Secondly, latex solution was added to AgNO_3_ solution and heated at 80 °C for 30 to 45 min and silver-latex nanoparticles were obtained gradually [57,121].

### 4.3. Characterization of Silver-Latex Nanoparticles

UV–visible spectral analysis was performed by detecting of the optical density (OD) using a “T80” UV/VIS spectrometer (Bruker Corporation, Billerica, MA, USA). Measurements were performed at room temperature between 200 and 800 nm ranges. The baseline was established by using silver nitrate (1 mM) as a blank. Transmission electron microscopy (JEOL JEM-2100 instrument, (JEOL Ltd., Tokyo, Japan)) was utilized to explore the morphology and size of silver nanoparticles. The sample was equipped by bringing a drop of them on a carbon-coated copper grid and using a lamp to dry it.

Fourier transform infrared (FTIR) spectroscopy measurements were used to confirm the AgNPs synthesis and also to estimate the possible bioactive components in the plant latex that enhance the reduction of the Ag^+^ ions and play roles in stabilization of the synthesized nanoparticles [122]. Both crude latex and silver nanoparticle samples were ground to dry semisolid form and mixed with Kbr and analyzed using a Nicolet^TM^ iS^TM^ 10 FTIR spectrometer (Thermo Scientific, Inc., Waltham, MA, USA). The results were detected in the range of 4000–400 cm^−1^ at a resolution of 8 cm^−1^ at 25°C.

### 4.4. Phytochemical Analysis

#### 4.4.1. Total phenolic Contents

The total phenol components were evaluated using the Folin-Ciocalteu procedure improved by Wolfe et al. [123] and Issa et al. [124] (that included using of gallic acid as a standard). The total phenolic constituents in the latex samples were quantitated as equivalents in milligrams of gallic acid/dried plant latex extract in grams concerning the standard curve (y = 0.0062x, r^2^ = 0.987).

#### 4.4.2. Total Flavonoid Contents

Flavonoids in the studied taxa’s latex were valued by a colorimetric estimation using aluminum chloride [125] and catechin as a standard. The calculated values of flavonoid constituents were quantified as equivalents of catechin in milligram per dried latex samples in grams regarding the standard curve (y = 0.0028 x, r^2^ = 0.988).

#### 4.4.3. Total Tannin Contents

Estimation of the total tannin components in plant latex was performed using a vanillin-hydrochloride assay [126,127] and the resulting values of the samples were quantified as equivalents of tannic acid in grams/gram dry sample.

#### 4.4.4. Total Alkaloid Contents

Fifty mL of 10% acetic acid in ethanol was added to 1 g of the sample and covered, then allowed to stand for 4 h. After that, it was filtered and the sample was concentrated in a water bath. Concentrated ammonium hydroxide was then added on top wisely to the sample till the precipitation was finished. The solution was allowed to settle and the precipitate obtained, then it was washed using diluted ammonium hydroxide. Finally, it was filtered and dried to a constant weight [128].

#### 4.4.5. Determination of Heavy Metals in Latex

a. Acid digestion

About 0.4 gm of each plant latex was taken to be digested using 8 mL of concentrated sulfuric acid in the presence of (2.14 gm) digestion mixture [1 kg potassium sulphate and 60 gm of mercuric oxide (red)] [129].

b. Atomic absorption spectrophotometer analysis

The prepared aliquot mixtures were used to estimate the concentration of cadmium (Cd), cobalt (Co), cobber (Cu) and iron (Fe) using an atomic absorption spectrophotometer (Buck Scientific Accusys 211 series, USA) by an air/acetylene flame system. The concentration of metals in each latex sample was estimated in mg/L [130]. 

### 4.5. GC–MS of C. Acutum Latex

Latex constituents of *C. acutum* were screened using GC-MS-QP2010 Ultra analysis equipment (Shimadzu Europa, Duisburg, Germany). The oven temperature was started at 50 °C, held for 3 min, then rose by 8 °C/min to 250 °C and held for 10 min. In electron impact mode, the spectrophotometer was used. The injector, interface, and ion source were maintained at 250, 250, and 220 °C, respectively. Helium served as the carrier gas for the split injection, which used a split ratio of 1:20 and a column SLB-5ms (silphenylene polymer, virtually equivalent to poly (5% diphenyl/95% methylsiloxane)) flow rate of 1.5 mL/min to inject a 1 µL diluted sample in *n*.hexane (1:1, *v*/*v*). The main single components were identified using WILEY and National Institute of Standards and Technology (NIST08) libraries based on their relative indices and mass spectra.

### 4.6. Antioxidant Activity

By observing the disappearance of DPPH at 520 nm, antiradical activity was quantified spectrophotometrically using a UV-visible spectrophotometer. The reaction mixtures for each treatment were made up of 3.9 mL of 0.1 mM DPPH dissolved in ethanol and 100 µL of supernatant. All treatments were incubated at room temperature for 30 min. Each treatment was measured three times. The sample without an antioxidant served as the control, while ethanol was utilized as a blank. The DPPH activity was expressed as a percentage of inhibition and calculated using the following Equation [131]:

$$\mathrm{Inhibition}\;\%=\left(1-\frac{AS}{AB}\right)\times \;100$$

where *AB* = absorbance of control sample (*t* = 0 h) and *AS* = absorbance of a tested sample after the reaction (*t* = 1 h).

### 4.7. Antibacterial Activity of Cy-AgNPs

The antibacterial activity of C. acutum latex 3% extract and different Cy-AgNP concentrations (25 mg/L and 50 mg/L) against *Bacillus subtilis*, *Escherichia coli*, and *Staphylococcus aureus* bacterial strains in vitro were compared with 10 g gentamicin standard antibiotics per 5 mm paper disc, using the disc diffusion method [132].

### 4.8. Determination of Cytotoxicity Using Chromosomal Aberration Assay (Vicia faba Test)

#### 4.8.1. Pre-Treatment

Seeds of *Vicia faba* L. (var. Giza 3) were obtained from the National Gene Bank, Ministry of Agriculture and Land Reclamation. The seeds were drowned in distilled water at 26 °C for 24 h and germinated in Petri dishes between two layers of cotton. Roots of 1.5–2.0 cm in length were used to examine the effect of latex and different concentrations of silver nanoparticles.

#### 4.8.2. Preparation of Silver-Latex Nanoparticles

Immediately after synthesis and characterization of Ag-NPs, they were suspended in deionized water and dispersed using ultrasonic vibration (100 W, 30 KHz) for 30 min in order to prepare three different concentrations at 25 mg/L, 50 mg/L, and 100 mg/L. *V. faba* seeds were treated with different latex nanoparticle concentrations in addition to crude latex (3%); untreated samples (control) were operated using distilled H_2_O. Samples were coded per data of Table 6.

#### 4.8.3. Fixation of Roots, Slide Preparation, and Microscopic Examination

**Figure 14:**
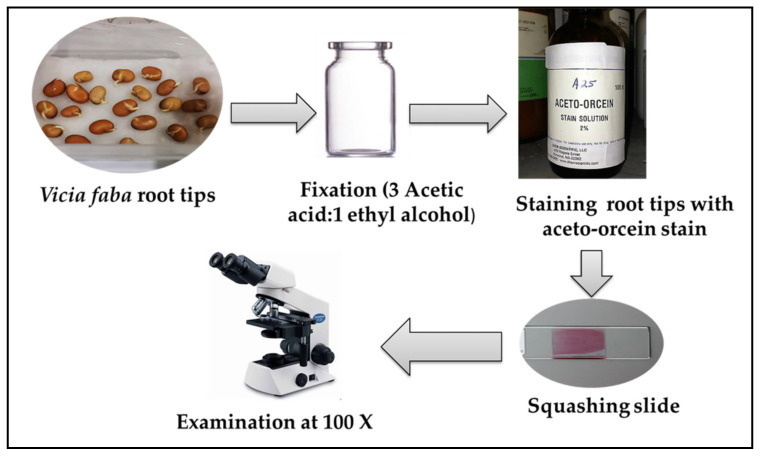
Diagrammatic scheme for preparation of chromosomal aberrations slide.

After treatment of *V. faba* seeds for 24 h, the preparation of slides was demonstrated in Figure 14 and illustrated as follows: The root tips of bean seeds were fixed in glacial acetic acid/ethanol with ratio 1:3 (Carney’s solution) and stored in a refrigerator for at least 48 h or until use. Roots were soaked in distilled water for 5 min for washing and then hydrolyzed in 1N HCl at 60 °C for 6–8 min. Next, the root tips were rinsed in water and stained by an aceto-orcein stain [133] for 2–4 h to prepare a slide. The dark stained root tips were erased in one drop of 45% acetic acid on a clean slide and squashed under a cover glass to disperse the cells. Electric microscope (Olympus CX 40) was used to record normal and aberrant cells which were registered in different stages of mitosis.

### 4.9. Data Analysis

The cytotoxic potential was studied by demonstrating of the mitotic index (MI), phase indices (PI), and total abnormality percentage at different phases of cell division. The data were statistically analyzed using *t*-tests in order to estimate the alteration among different treatments and the untreated sample [134].

### 4.10. Biochemical Study (Protein SDS-PAGE)

Polyacrylamide gel electrophoresis in the presence of sodium dodecyl sulphate (SDS-PAGE) was operated to obtain proteins electrophoretic profiles of treated *V. faba* seeds in order to estimate the genotoxic effect of latex extract and different concentrations of its silver nanoparticles. The method for the discontinuous SDS-PAGE technique was based on that of Laemmli [135] and modified by Studier [136].

### 4.11. Molecular Study (ISSR Marker)

Eight primers were tested to amplify the isolated DNA from treated *V. faba*. Table 7 shows the primers and their sequences. Extraction of DNA was done using EZ-10 spin Column genomic DNA minipreps kit handbook (plant) (BIO BASIC INC.).

**Table 7:** Sequences and codes of eight ISSR primers.

Primer	Sequence
ISSR-1	GAGAGAGAGAGAGAGAC
ISSR-2	CACACACACACACACAG
ISSR-5	CACACACACACAGG
ISSR-10	(GA)7GT
ISSR-11	(GACA)4
ISSR-12	T(GA)9
ISSR-14	(CTCT)4GTC
HB-14	GAGGAGGAGGC

### 4.12. Estimation of Genomic Template Stability (GTS%)

Genotoxicity was observed in the SDS-PAGE and ISSR profiles by recording disappearance of normal bands and appearance of new bands. Only clear and reproducible bands were observed in order to assess any disorder in DNA and demonstrate the genomic template stability percentage (GTS%). Polymorphisms recorded in the SDS-PAGE and ISSR profiles included disappearance of a normal band and appearance of a new band compared with the control profile [137].

The GTS% was calculated for each sample of treatments according to the formula of Sukumaran and Grant [55] as:

$$\mathrm{GTS}\;=\;1-\frac{a}{n}\times 100$$

where “a” indicates the polymorphic profiles in each sample and “n” is the number of total bands in the control.

## 5. Conclusions

This study was conducted mainly to investigate the effect of different concentrations of silver nanoparticles from *C. acutum* latex and its crude latex on biochemical and molecular DNA level and mitotic division using *Vicia faba* seeds. The reducing effect of the MI% was clearly observed by increasing Cy-AgNP concentrations (50 and 100 mg/L, where Cy-AgNPs (25 mg/L) treatment showed moderate decrease in MI% compared to *C. acutum* latex (3%) and control. Generally, all treatments showed increasing chromosomal abnormalities, but Cy-AgNPs (25 mg/L) expressed the lowest percentage, and by increasing the concentration of AgNPs, the percentage increased. Genomic template stability percentage (GTS%) by using biochemical protein SDS-PAGE and molecular ISSR markers showed the highest GTS% in the 25 mg/L Cy-AgNPs treatment (80% in SDS-PAGE and 51.28% in ISSR marker). Finally, this paper concluded that the 25 mg/L Cy-AgNPs have the highest content of bioactive constituents (TPC, TFC, tannins, and alkaloids) and showed lowest cytotoxicity and genotoxicity. The highest antioxidant activity using the DPPH method was reported in Cy-AgNPs (25 mg/L) (70.26 ± 1.32%). The highest antibacterial activity was for Cy-AgNPs (50 mg/L) against *Bacillus subtilis*, *Escherichia coli*, and *Staphylococcus aureus*. Chemical characterization of GC-MS revealed that n-alkanes such as tetradecanoic acid and hexadecanoic acid had the highest antimicrobial effect in addition to the presence of lupeol’s effect on the antioxidant activity of the studied latex. The use of AgNPs in low concentrations increased MI%, which can stimulate plant growth and development. Increasing the use of Cy-AgNPs in high concentrations leads to the opposite result of increasing chromosomal abnormalities and reducing GTS%. Therefore, uses of nanoparticles must be under strict supervision by health authorities, with limits to concentrations to reduce risks to human populations. 

## Figures and Tables

**Table 1:** The list of main constituents present in *C. acutum latex* using GC-MS analysis.

Quantitative ID	Component Identified	Retention Time (min)	Retention Index (RI)	Area (%)	Identification
**1**	Octanoic Acid	10.6	1015	3.62	RI, MS *
**2**	L-Glucose, 6-deoxy-3-Omethyl-	12.3	236	3.15	RI, MS
**3**	Octanal	13.5	874	2.99	RI, MS
**4**	Hexanoic acid	16. 9	963	2.67	RI, MS
**5**	Thymol	17.21	1290	2.11	RI, MS
**6**	Curcumene	18.44	1483	1.99	RI, MS
**7**	Elemenone	19.56	1593	2.86	RI, MS
**8**	Germacrone	20.22	1693	4.45	RI, MS
**9**	Lupeol	20.56	3270	15.36	RI, MS
**10**	tetradecanoic acid	21.12	1761	6.56	RI, MS
**11**	Hexadecanoic acid (palmitic acid)	21.73	2023	10.72	RI, MS
**12**	Phytol	21.95	2111	6.51	RI, MS
**13**	Octadecanoic acid	23. 42	2102	8.78	RI, MS
**14**	Naphthalene,	24.38	1179	5.21	RI, MS
**15**	7-nonenamide	24.65	2523	3.91	RI, MS
**16**	1-Octadecyne	41.71	1793	4.98	RI, MS
**17**	Neophytadiene	42.56	1827	9.15	RI, MS
**18**	Cis-Vaccenic acid	44.65	2141	1.78	RI, MS
**19**	Glycerol-1-Palmitate	45.67	3734	1.66	RI, MS
**20**	Tridecanal	45.87	1510	1.54	RI, MS

(*): RI, Retention index; MS: Mass Spectroscopy.

**Table 2:** Percentage of total abnormalities, normal and abnormal phase indices, and mitotic index for the treated *V. faba* root tips with 3% *C. acutum* latex and different concentrations of its silver nanoparticles.

Treatment		%MI	Phase Index								% Total Abnormal	
			% Prophase		% Metaphase		% Anaphase		% Telophase		Interphase	Mitosis
Samples	Conc.		Mitotic	Abn.	Mitotic	Abn.	Mitotic	Abn.	Mitotic	Abn.		
Untreated sample		10.70 ± 0.45	22.76	0.00	69.88	16.77	0.51	2.47	6.75	1.56	0.03 ± 0.02	20.81 ± 2.09
Latex of *C.acutum* L.	3% Crude Latex	7.98 ± 0.49 ns	20.81	0.00	79.19	25.96	0.00	0.00	0.00	0.00	0.02 ± 0.02	25.96 ± 2.87 *
	Cy-AgNPs 25 mg/L	9.08 ± 0.59 ns	15.67	0.00	49.04	15.73	9.73	2.25	25.56	5.43	0.16 ± 0.11	23.41 ± 3.90 *
	Cy-AgNPs 50 mg/L	4.95 ± 0.52 ns	1.64	0.00	84.72	55.82	3.64	3.64	10.00	7.28	0.93 ± 0.32	66.73 ± 6.82 ns
	Cy-AgNPs 100 mg/L	4.04 ± 0.30 ns	5.48	0.00	91.13	62.20	1.69	3.39	1.69	2.54	0.53 ± 0.15	68.14 ± 5.40 ns

* = significant at significant differences between different treatments at *p* ≤ 0.05; ns = No significant differences between different treatments at *p* ≤ 0.05.

**Table 3:** Effect of 3% *C. acutum* crude latex and different concentrations of Cy-AgNPs on protein profiles of *V. faba* and percentage of genomic template stability (GTS%) of the treated *V. faba* (P: polymorphic bands and M: monomorphic bands).

No.		Treatments					Polymorphism
		Control	3% *Cynanchum* Latex	25 mg/L Cy-AgNPs	50mg/L Cy-AgNPs	100mg/L Cy-AgNPs	
1	100	1	1	1	1	1	M.
2	90	1	1	1	1	1	M.
3	70	1	1	1	1	1	M.
4	60	1	1	1	1	1	M.
5	50	1	1	1	1	1	M.
6	40	1	1	1	1	1	M.
7	30	1	1	1	1	1	M.
8	28	1	1	1	0	0	P.
9	23	0	0	1	1	1	P.
10	20	1	1	1	1	1	M.
11	19	1	1	1	1	0	P.
12	18	1	1	1	0	0	P.
13	17	1	0	0	0	0	P.
14	15	1	1	1	1	1	M.
15	10	1	1	0	1	0	P.
16	5	1	1	1	1	1	M.
Total bands		15	14	14	13	11	
Polymorphic bands %		33	29	29	23	9	37.5%
GTS%		100	75.33%	80%	73.33%	60%	--------

**Table 4:** Total number of the amplified DNA bands, their type and the percentage of the total polymorphism resulted from eight ISSR primers in the treated *V. faba* seeds with 3% *C. acutum* latex and different concentrations of its silver nanoparticles.

Primer	Monomorphic Bands	Polymorphic Bands		Total Bands	Polymorphism %
		Unique Bands	Non-Unique Bands		
ISSR 1	1	1	8	10	90
ISSR 2	3	3	3	9	66.67
ISSR 5	2	1	6	9	77.78
ISSR 10	4	2	4	10	60
ISSR 11	3	0	3	6	50
ISSR 12	7	1	2	10	30
ISSR 14	1	4	9	14	92.86
HB 14	5	0	6	11	54.5
Total	23	12	44	79	70.89

**Table 5:** Effect of 3% *C. acutum* crude latex and different concentrations of its silver nanoparticles on ISSR profiles of *V. faba* and GTS% of the treated *V. faba*. a: refers to appearance of new bands, b: disappearance of normal bands and a + b: number of polymorphic bands.

Primer Name	Control	3% Latex		25 ppm AgNPs		50 ppm AgNPs		100 ppm AgNPs	
		a	b	a	b	A	b	a	b
ISSR 1	1	5	0	0	0	7	0	7	0
ISSR 2	5	3	0	1	0	1	2	1	2
ISSR 5	4	1	1	2	1	2	1	5	1
ISSR 10	4	0	0	4	0	3	0	6	0
ISSR 11	3	3	0	3	0	3	0	1	0
ISSR 12	10	0	2	0	1	0	2	0	2
ISSR 14	7	4	5	3	1	5	1	3	1
HB 14	5	5	0	4	0	6	0	6	0
Total number of bands	39	21	8	17	3	27	6	29	6
a + b	-----	29		20		33		35	
GTS%	100%	25.64%		51.28%		15.38%		10.26%	

**Table 6:** List of extracts concentrations and their codes.

Treatments	Code
Control	1
Crude latex of *C. acutum* L. (3%)	2
Silver nanoparticles of *C. acutum* L. (25 Cy-AgNPsmg/L)	3
Silver nanoparticles of *C. acutum* L. (50 Cy-AgNPs mg/L)	4
Silver nanoparticles of *acutum* L. (100 Cy-AgNPs mg/L)	5

## Data Availability

Relevant data applicable to this research are within the paper.
